# Supplementary material for: trans-α-Necrodyl Acetate: Minor Sex Pheromone Component of the Invasive Mealybug Delottococcus aberiae (De Lotto)
Source: Insects. 2025 Mar 19;16(3):318. doi: 10.3390/insects16030318 (PMC11943407; doi:10.3390/insects16030318)

Supporting Information

Manuscript title: *trans*-α-Necrodyl acetate: minor sex pheromone component of the invasive mealybug *Delottococcus aberiae* (De Lotto)

- **General procedures of synthesis**
- **Figure S1.** Synthesis of racemic *cis*-α-necrodyl acetate
- **Protocol S1**. Synthesis of racemic cis-α-necrodyl acetate
- **Figure S2.** Synthesis of racemic *trans*-α-necrodyl acetate
- **Protocol S2**. Synthesis of racemic *trans*-α-necrodyl acetate
- **Protocol S3.** Purification of the (1*R*,4*R*)-*trans*-α-necrodyl acetate [(1*R*,4*R*)-2] isolated from *Lavandula stoechas* subp. *luisieri* essential oil.
- **^{1}H NMR and ^{13}C NMR spectra** of the synthetic compounds.
- **Figure S3.** Mass spectra fragmentation observed for samples of (*trans*)-α-necrodol obtained by hydrolysis of the minor sex pheromone component of D. aberiae virgin female volatile collections (A), synthetic *β*-necrodols (±)-(*cis*)-3 or (±)-(*trans*)-3 (B) and γ-necrodol (C).
- **Figure S4.**Gas chromatography (GC) with chiral stationary phase column of (1*R*, 4*R*)-6, (±)-(*cis*)-6 and (±)-(*trans*)-6.

General Procedures of synthesis. The ^1H and ^{13}C spectra were recorded on a Bruker AC-300 spectrometer (Bruker, Billerica, MA) using $CDCl_3$ or C_6D_6 as the solvent. Chemical shift values in ^1H and ^{13}C NMR are reported in δ (ppm) relative to residual chloroform (7.26/77.0 ppm) or benzene (7.16/128.4 ppm). High-resolution mass spectra (ESI-HRMS) were measured on a Waters Xevo Q-TOF spectrometer (Waters Corp., Milford, MA) coupled with an Acquity UPLC-PDA system (Waters Corp., Milford, MA) using ionization by electrospray (ESI). The ESI source operated in the positive ionization mode using leucine-enkephalin as the reference mass ($[M+H]^+$ ion *m/z* 556.2771). The sample (2 µL) was injected into a Waters Acquity BEH column (50 × 2.1 mm i.d., 1.7 µm) using MeOH as an isocratic eluent. The GC-MS analyses were performed with the aforementioned equipment (apparatus and column) and the following oven temperature program: 55 °C for 3 min, raised at 15 °C/min up to 180 °C and then at 35 °C/min up to 280 °C, held for 6 min. A helium flow of 1 mL/min and an injection volume of 1 µL were employed. Detection and spectral acquisition were performed as indicated above.

All reagents and solvents (reagent grade) were purchased from Sigma-Aldrich (Madrid, Spain) and employed with no further purification unless otherwise stated. In the case of reactions requiring anhydrous conditions, the solvents used were dried with the appropriate drying agents and distilled before use. Unless otherwise noted, all reactions sensitive to moisture and/or air were carried out under a nitrogen atmosphere. The solvent extracts of the reaction mixtures were dried over anhydrous $MgSO_4$ and concentrated by rotary evaporation under reduced pressure. Crude products were purified by column flash chromatography using silica gel Merck 9385 (230−400 mesh). Thin-layer chromatography (TLC) was performed using Macherey-Nagel silica gel 60 F254 plates with a fluorescent indicator and UV light of 254 nm wavelength as the visualizing agent. Ceric ammonium molybdate and *p*-anisaldehyde were used as stains.

Figure S1. Synthesis of racemic *cis*-α-necrodyl acetate.

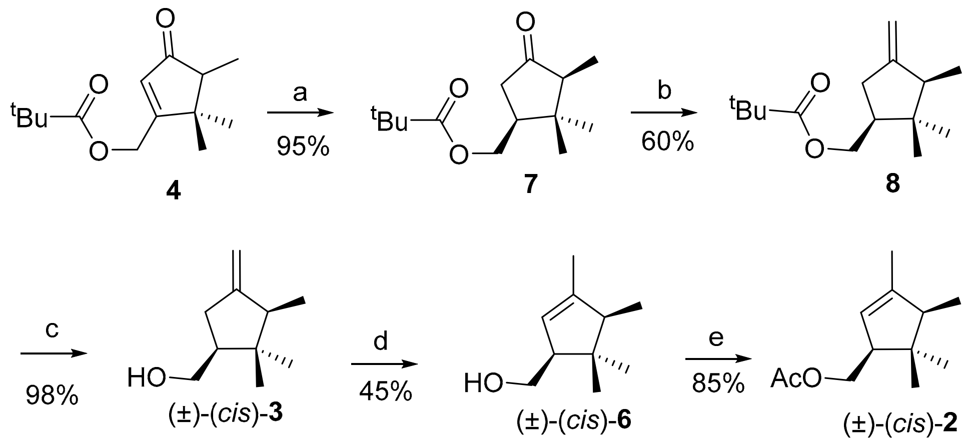

Synthesis of (±)-*cis*-*α*-necrodyl acetate, (±)-(*cis*)-**2**, from racemic enone **4**. *Reagents and conditions*: a) H_2, Pd/C, EtOAc, 5h; b) MeMgCl, Cp_2TiCl_2, 90ºC, 4h; c) K_2CO_3, MeOH, 40 ºC, 24 h; d) Li, $NH_2CH_2CH_2NH_2$, 90 ºC, 10 min; e) Ac_2O, Et_3N, DMAP, CH_2Cl_2, rt, 4h; All compounds are racemic, for clarity, only one enantiomer is drawn.

Protocol S1. Synthesis of racemic *cis*-*α*-necrodyl acetate. The synthesis of the diastereomer (±)-(*cis*)-**2** started with the diastereoselective hydrogenation of cyclopentenone **4** using Pd on carbon as a catalyst to afford saturated cyclopentenone **7** in 95% yield (Figure 1), obtained in very high diastereomeric pure form, as deduced from the analysis of the ^{1}H-NMR spectra. Methylenation of the carbonyl group, using Petasis's reagent, gave the *cis*-*β*-necrodol pivalate ester **8** in a moderate 60% yield, which after treatment with methanol under basic conditions afforded *cis*-*β*-necrodol (±)-(*cis*)-**3**, in nearly quantitatively yield. Using the previously described conditions for the isomerization of *β*-necrodol with lithium in ethylenediamine, *cis*-*β*-necrodol **3** led to a 5:1:3 mixture of *cis*-*α*-, *cis*-*β*-, and *γ*-necrodols, from which *cis*-*α*-necrodol **6** could be isolated in 45% yield after careful column chromatography. Acetylation of the hydroxyl group of (±)-(*cis*)-**6** under standard conditions using acetic anhydride and triethylamine as base afforded a pure sample of (±)-*cis*-*α*-necrodyl acetate [(±)-(*cis*)-**2**] in 85 % yield.

rac-(cis-2,2,3-Trimethyl-4-oxocyclopentyl)methyl pivalate (**7**). Palladium on carbon (Pd/C, 5 wt.%) (60 mg) was added to a solution of racemic enone **4** (600 mg, 2.5 mmol) in EtOAc (60 mL) in a 100 mL Pyrex round-bottom flask. The system was then evacuated and backfilled with H_2 gas from a balloon in cycles. The reaction mixture was stirred at room temperature for 6 hours, and then filtered through silica gel, using EtOAc as eluent, to give 570 mg (95%) of ketone **7** as a yellowish oil that required no further purification. ^{1}H NMR (300 MHz, $CDCl_3$) δ 4.25 (1H, dd, *J* = 11.2, 6.1 Hz, CHO), 4.09 (1H, dd, J = 11.2, 7.6 Hz, CH'O), 2.46 (1H, ddd, J = 18.9, 8.4, 1.3 Hz, H-5), 2.33 - 2.22 (1H, m, H-1), 2.04 (1H, qd, *J* = 7.0, 1.3 Hz, H-3), 1.97 (1H, dd, J = 18.9, 11.5, H'-5), 1.21 (3H, s, Me-2), 1.20 (9H, s, *t*-Bu), 0.95 (3H, d, *J* = 7.0 Hz, Me-3), 0.70 (3H, s, Me'-2); ^{13}C NMR (75 MHz, $CDCl_3$) δ 217.8 (C-4), 178.6 (CO_2), 64.4 (CH_2O), 57.2 (C-3), 44.2 (C-

1), 41.0 (C-2), 39.2 (C-5), 38.9 (Me_3*C*), 27.3 (*Me_3*C), 26.9 (Me-2), 16.1 (Me'-2), 7.1 (Me-3); HRMS (TOF MS ESI+) calcd for $C_{14}H_{25}O_3$ $[M+H]^{\cdot+}$ 241.1798, found 241.1786.

rac-(cis-2,2,3-Trimethyl-4-methylenecyclopentyl)methyl pivalate (**8**). A 3M solution of methylmagnesium chloride in THF (1.2 mL, 3.6 mmol) was added to a suspension of *bis*(cyclopentadienyl)titanium(IV) dichloride (453 mg, 1.8 mmol) in toluene (12 mL) at 0 °C. After 40 minutes of stirring, the mixture was gradually warmed to room temperature, and a solution of ketone **7** (220 mg, 0.91 mmol) in dry toluene (1 mL) was added. The solution was heated to 90 °C for 4 hours, cooled to room temperature, and the solvent evaporated under reduced pressure. The residue obtained was directly purified by flash column chromatography on silica gel, using a 95:5 mixture of hexane and Et_2O as eluent, to yield 133 mg (60% yield) of *cis*-β-necrodyl pivalate **8** as a yellowish oil. ^{1}H NMR (300 MHz, $CDCl_3$) δ 4.84 (1H, m, =CH), 4.75 (1H, qd, J = 2.5, 1.0 Hz, =CH'), 4.13 (1H, dd, J = 10.9, 6.2 Hz, CHO), 4.00 (1H, dd, J = 10.9, 7.4 Hz, CH'O), 2.65-2.52 (1H, m, H-5), 2.08-1.91 (3H, m, H-1, H'-5 and H-3), 1.20 (9H, s, *t*-Bu), 1.06 (3H, s, Me-2), 0.93 (3H, d, J = 6.8 Hz, Me-3), 0.56 (3H, s, Me'-2); ^{13}C NMR (75 MHz, $CDCl_3$) δ 178.6 (CO_2), 154.6 (C-4), 104.7 (=CH_2), 65.6 (CH_2O), 50.6 (C-3), 46.8 (C-1), 42.4 (C-2), 38.9 (Me_3*C*), 34.2 (C-5), 27.4 (*Me_3*C), 26.4 (Me-2), 15.1 (Me'-2), 10.6 (Me-3); HRMS (TOF MS ESI+) calcd for $C_{15}H_{27}O_2$ $[M+H]^{\cdot+}$ 239.2006, found 239.2002.

rac-(cis-2,2,3-Trimethyl-4-methylenecyclopentyl)methanol ((±)-(*cis*)-**3**). Potassium carbonate (116 mg, 0.84 mmol) was added to a solution of **8** (100 mg, 0.42 mmol) in MeOH (8 mL). The suspension was stirred for 8 h at 45ºC. After this time, water (10 mL) was added, and the solution was extracted with EtOAc (2x10 mL). The combined organic layers were washed with brine (10 mL), dried over anhydrous $MgSO_4$, and concentrated under reduced pressure. The crude residue was purified by flash column chromatography on silica gel, using a 7:3 mixture of hexane and EtOAc

as eluent, to give 63 mg (98 % yield) of *cis*-β-necrodol, (±)-(*cis*)-**3**, as a yellowish oil. Its spectroscopic data were fully coincident with those previously described in the literature [27].

rac-(cis-3,4,5,5-Tetramethylcyclopent-2-enyl)methanol ((±)-(*cis*)-**6**). *cis*-β-Necrodol **3** (100 mg, 0.65 mmol) was added to a solution of Li (100 mg, 14 mmol) in anhydrous ethylenediamine (2.5 mL) heated at 70ºC. The solution was stirred for 10 min and then, directly poured into ice. The mixture was extracted with Et_2O (2 × 10 mL), and the combined organic layers were successively washed with a saturated aqueous solution of NH_4Cl (6 mL) and brine (6 mL), dried over anhydrous $MgSO_4$, and concentrated under reduced pressure. The crude residue obtained was purified by flash column chromatography on silica gel, using a 7:3 mixture of hexane and Et_2O as eluent, to give in order of elution 45 mg (45% yield) of *cis*-α-necrodol **6**, 9 mg (9% yield) of *cis*-β-necrodol **3** and 27 mg (27% yield) of *γ*-necrodol, all of them as a yellowish oil. The spectroscopic data for all three were in agreement with those previously reported [28].

rac-(cis-3,4,5,5-Tetramethylcyclopent-2-enyl)methyl acetate [(±)-(*cis*)-**2**]. *cis*-α-Necrodol **6** (40 mg, 0.26 mmol) was dissolved in CH_2Cl_2 (15 mL), and triethylamine (90 μL, 0.65 mmol), catalytic 4-dimethylaminopyridine (DMAP), and anhydride acetic (50 μL, 0.52 mmol) were added at room temperature. The solution was stirred for 2 h and then poured into water (10 mL). The mixture was diluted with more CH_2Cl_2 (15 mL). The organic layer was successively washed with 1M aqueous HCl (8 mL), saturated aqueous $NaHCO_3$ (8 mL), and brine (8 mL), and dried over anhydrous $MgSO_4$. The crude residue

obtained after evaporation of the solvent under reduced pressure was purified by flash column chromatography on silica gel, using a 98:2 mixture of hexane and Et_2O as eluent, to give 43 mg (85% yield) of (±)-(*cis*)-**2** as a yellowish oil. ^{1}H NMR (400 MHz, $CDCl_3$) δ 5.17 (1H, dd, *J* = 3.4, 1.7 Hz, H-2), 4.05 (1H, dd, *J* = 10.8, 7.0 Hz, CHO), 3.95 (1H, dd, *J* = 10.8, 7.5 Hz, CH'O), 2.56 - 2.42 (1H, m, H-1), 2.23 – 2.08 (1H, m, H-4), 2.04 (3H, s, $MeCO_2$), 1.65 (3H, dt, *J* = 2.4, 1.4 Hz, Me-3), 1.08 (3H, s, Me-5), 0.89 (3H, d, *J* = 7.4 Hz, Me-4), 0.77 (3H, s, Me'-5); ^{13}C NMR (75 MHz, $CDCl_3$) δ 171.4 (CO_2), 144.8 (C-3), 123.2 (=CH_2), 65.8 (CH_2O), 53.7 (C-1), 53.0 (C-4), 43.8 (C-5), 29.6 (Me-5), 21.2 (*Me*CO_2), 18.1 (Me'-5), 15.2 (Me-3), 13.3 (Me-4); MS (70eV) m/z 41 (22), 43 (48), 55 (9), 69 (10), 77 (6), 121 (100), 122 (10), 123 (16), 136 (17), 154 (0.1).

Figure S2. Synthesis of racemic *trans*-α-necrodyl acetate.

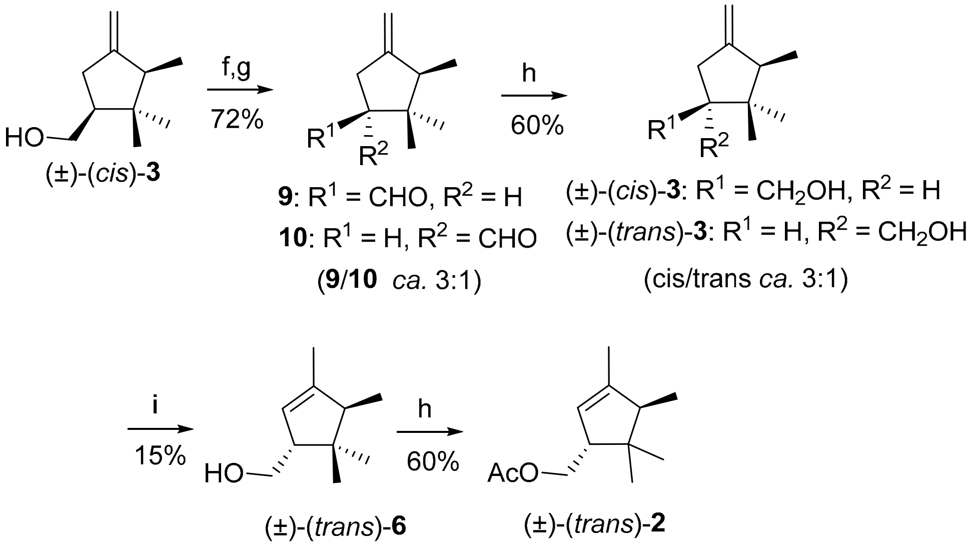

Synthesis of (±)-*trans*-*α*-necrodyl acetate, (±)-(*trans*)-**2**. *Reagents and conditions*: f) Dess-Martin periodinane, CH_2Cl_2, rt, 1 h; g) NaOMe, Et_2O, 0 ºC, 5 h; h) $NaBH_4$, MeOH, rt, 3h; i) Li, $NH_2CH_2CH_2NH_2$, 90 ºC, 10 min, then column chromatography (see text). All compounds are racemic, for clarity, only one enantiomer is drawn.

Protocol S2. Synthesis of racemic *trans*-α-necrodyl acetate. The preparation of the diastereomer (±)-(*trans*)-**2** was carried out as follows. *cis*-*β*-Necrodol, (±)-(*cis*)-**3**, was oxidized to the corresponding *cis*-*β*-necrodal **9** using Dess-Martin periodinane as an oxidant reagent in 85 % yield. Treatment of **9** with sodium methoxide at 0 ºC, under strictly controlled time and temperature conditions, afforded a *ca*. 3:1 mixture of *cis*- and *trans*-*β*-necrodals **9** and **10**, respectively, as determined by GC-FID and ^{1}H NMR spectroscopy. The mixture of epimeric necrodals was reduced using sodium borohydride in methanol to obtain a *ca*. 3:1 mixture of diasteromeric *cis*- and *trans*-*β*-necrodol, (±)-(*cis*)-**3** and (±)-(*trans*)-**3**, respectively. Since conventional chromatographic procedures could not separate these *β*-necrodols, the mixture of both was directly subjected to the double bond isomerization conditions previously used for the isomerization of (±)-(*cis*)-**3** to (±)-(*cis*)-**6.** This reaction provided a mixture of partial isomerization products of the exocyclic double bond to endocyclic positions, consisting in a mixture of *cis*- and *trans* *α*-and *β*-necrodols and *γ*-necrodol, in a proportion of 5:1:3 respectively, as deduced from the analysis of its ^{1}H NMR spectrum. Careful flash column chromatography of this mixture allowed to obtain a pure sample of *trans*-*α*-necrodol, (±)-(*trans*)-**6**, although with a very low yield (15%) but in sufficient quantity for its spectroscopic characterization and the objective pursued. Standard acetylation of (±)-(*trans*)-**6** using acetic anhydride and triethylamine as base afforded a pure sample of (±)-*trans*-*α*-necrodyl acetate [(±)-(*trans*)-**2**] in 81% yield.

rac-cis-2,2,3-Trimethyl-4-methylenecyclopentane-1-carbaldehyde (**9**). A 0.3M solution of the Dess-Martin periodinane reagent in CH_2Cl_2 (1.08 mL, 0.32 mmol) was added to a solution of *cis*-β-necrodol **3** (42 mg, 0.27 mmol) in CH_2Cl_2 (8 mL) at room temperature. After stirring at this temperature for 1 h, the solution was washed with a saturated aqueous solution of $NaHCO_3$ (5 mL) and brine (5 mL) and dried over anhydrous $MgSO_4$. The residue obtained after evaporation of the solvent under reduced pressure was purified by flash column chromatography on silica gel, using an 8:2 mixture of hexane and Et_2O as eluent, to yield 34 mg (83% yield) of aldehyde **9**. ^1H NMR (300 MHz, $CDCl_3$) δ 9.79 (1H, d, J = 2.2 Hz, CHO), 4.93 (1H, ddd, J = 3.9, 2.4, 0.6 Hz, CH'H-4), 4.81 (1H, td, J = 2.9, 0.6 Hz, CHH'-4), 2.86 – 2.65 (1H, m, H-5), 2.60 – 2.40 (2H, m, H-1 and H'-5), 2.28 – 2.07 (1H, m, H-3), 1.24 (3H, s, Me-2), 0.94 (3H, d, J = 6.8 Hz, Me-3), 0.66 (3H, s, Me'-2); ^{13}C NMR (75 MHz, $CDCl_3$) δ 204.5 (CHO), 153.3 (C-4), 105.8 (=CH_2), 60.1 (C-1), 50.9 (C-3), 45.0 (C-2), 29.6 (C-5), 26.6 (Me-2), 16.2 (Me'-2), 10.3 (Me-3); HRMS (TOF MS ESI+) calcd for $C_{10}H_{17}O$ $[M+H]^{+}$ 153.1274, found 153.1265.

rac-(trans-3,4,5,5-Tetramethylcyclopent-2-enyl)methanol (**10**). A solution of the *cis* aldehyde **9** (200 mg, 1.3 mmol) in Et_2O (30 mL) was treated with a 30% solution of sodium methoxide in methanol (300 μL) and stirred at 0 ºC for 5 h. After this time, the reaction mixture was successively washed with water (10 mL) and brine (10 mL) and dried over anhydrous $MgSO_4$. The solvent was distilled under reduced pressure to give 180 mg (90% yield) of a *ca*. 3:1 mixture of diastereomeric *cis*- and *trans*-aldehydes **9** and **10**, respectively, as a yellowish oil. The crude obtained mixture was used in the next step with no further purification.

Sodium borohydride (45 mg, 1.17 mmol) was added to a solution of the mixture of aldehydes **9** and **10** (180 mg, 1.17 mmol) in MeOH (18 mL) at 5 ºC. After stirring for 30 min, the reaction mixture was poured into water (10 mL) and extracted with

EtOAc (2x10 mL). The combined organic layers were washed with brine (10 mL) and dried over anhydrous $MgSO_4$. Evaporation of the solvent under reduced pressure afforded 172 mg (95%) of a *ca.* 3:1 mixture of diastereomeric *cis*- and *trans-β-necrodols* **3**, respectively**,** as a yellowish oil. The NMR spectroscopic data of the *trans* diastereomer, (±)-(*trans*)-**3**, deduced from the spectra of the mixture, was fully coincident with those previously described in the literature [28].

An obtained mixture of *cis*- and *trans-β*-necrodols **3** (172 mg, 1.12 mmol) was treated with a solution of Li (60 mg, 8.64 mmol) in anhydrous ethylenediamine (2.5 mL), as described previously for the transformation of (±)-(*cis*)-**3** into (±)-(*cis*)-**6.** The obtained mixture of isomerization products of the double bond (175 mg) was chromatographed on silica gel, using 7:3 mixture of hexane and Et_2O as eluent, to give, in order of elution, the *trans-α*-necrodol (±)-(*trans*)-**6** (24 mg, 14%), followed by a chromatographically homogeneous mixture (130 mg) of the (±)-(*cis*)-**6** and (±)-(*cis*)-**3***,* (±)-(*trans*)-**3***,* and *γ*-necrodols. The spectroscopic data of *trans-α*-necrodol (±)-(*trans*)-**6** were fully coincident with those previously described in the literature for this compound (See literature reference given for compound (±)-(*trans*)-**3**).

rac-(trans-3,4,5,5-Tetramethylcyclopent-2-enyl)methyl acetate [(±)-(*trans*)-**2**]. The diastereomeric *α*-necrodyl acetate (±)-(*trans*)-**2** (21 mg, 81% yield) was obtained from *trans-α*-necrodol (±)-(*trans*)-**6** (20 mg, 0.38 mmol) following a similar procedure to that described for the transformation of *cis-α*-necrodol (±)-(*cis*)-**6** into the acetate (±)-(*cis*)-**2**. Its spectroscopic data were coincident with those previously reported in the literature [14]. ^1H NMR (300 MHz, $CDCl_3$) δ 5.16 (1H, dd, J = 3.3, 1.6 Hz, H-2), 4.10

(1H, dd, J = 10.8, 6.6 Hz, CHO), 3.93 (1H, dd, J = 10.8, 7.5 Hz, CH'O), 2.52 - 2.44 (1H, m, H-1), 2.15 – 2.07 (1H, m, H-4), 2.04 (3H, s, $MeCO_2$), 1.67 – 1.64 (3H, m, Me-3), 0.95 (6H, s, 2xMe-5), 0.88 (3H, d, J = 7.3Hz, Me-4); ^{13}C NMR (75 MHz, $CDCl_3$) δ 171.4 (CO_2), 145.5 (C-3), 123.2 (=CH_2), 65.1 (CH_2O), 52.7 (C-1), 52.5 (C-4), 43.2 (C-5), 24.6 (Me-5), 24.1(Me'-5), 21.2(MeCO2), 15.3 (Me-3), 12.5 (Me-4); MS (70eV) m/z 41 (22), 43 (48), 55 (9), 69 (10), 77 (6), 121 (100), 122 (10), 123 (16), 136 (17).

Protocol S3. Purification of the (1*R*,4*R*)-*trans*-α-necrodyl acetate [(1*R*,4*R*)-2] isolated from *Lavandula stoechas* subp. *luisieri* essential oil.

((1R,4R)-3,4,5,5-tetramethylcyclopent-2-en-1-yl)methyl 4-nitrobenzoate (**5**). (1*R,*4*R*)-**2** (250 mg of 90% purity, 1.27 mmol) was treated with a 0.2M solution of potassium carbonate in MeOH (8 mL) and stirred for 1 h at room temperature. Then water (10 mL) was added, and the mixture was extracted with EtOAc (2x15 mL). The combined organic layers were washed with brine (10 mL) and dried over anhydrous $MgSO_4$. The solvent was removed under reduced pressure, and the resulting crude residue was dissolved in CH_2Cl_2 (15 mL). Triethylamine (0.35 mL, 2.55 mmol), a catalytic amounts of 4-dimethylaminopyridine (DMAP), and p-nitrobenzoyl chloride (330 mg, 1.78 mmol) were added at room temperature. The resulting solution was stirred for 2 h, then poured into water (10 mL) and extracted with CH_2Cl_2. The combined organic layers were successively washed with 1M aqueous HCl (5 mL), saturated aqueous $NaHCO_3$ (5 mL), and brine (5 mL), and dried over anhydrous $MgSO_4$. The crude residue obtained after evaporation of the solvent was purified by flash column chromatography on silica gel, using an 85:15 mixture of hexane and Et_2O as eluent, to give 310 mg (80% yield) of nitrobenzoate ester **5** as a white solid. This compound was crystallized from hexane as follows, (300 mg) was dissolved in 3.5 ml of hexane at 25°C with stirring. The solution was transferred to a vial and then cooled to -15°C for 24 hours. After filtering and washing with cold hexane, the crystals were dried under high vacuum (0.01 mbar). 200 mg (52% yield) of ester **5** was obtained as a white solid with a purity of 99% on GC. ^{1}H NMR (400 MHz, $CDCl_3$) δ 8.32 – 8.26 (2H, m, H-3 and H-5 Ar), 8.22 – 8.16 (1H, m, H-2 and H-6 Ar), 5.23 (1H, dd, *J* = 3.5, 1.7 Hz, H-2), 4.39 (1H, dd, J = 10.9, 6.7 Hz, CHO), 4.25 (1H, dd, J = 10.9, 7.0 Hz, CH'O), 2.70-2.59 (1H, m, H-1). 2.23 – 2.12 (1H, m, H-4), 1.67 (3H, dd, J = 1.7, 0.7 Hz, Me-3), 1.03 (3H, s, Me-5), 1.01 (3H, s, Me´-5), 0.91 (3H, d, *J* = 7.3

Hz, Me-4); ^{13}C NMR (75 MHz, $CDCl_3$) δ 164.9 (CO_2), 150.6 (C-Ar), 146.1 (C-3), 136.1 (C-Ar), 130.8 (C-Ar), 123.7 (C-Ar), 122.8 (=CH_2), 66.4 (CH_2O), 52.8 (C-1), 52.7 (C-4), 43.3 (C-5), 24.8 (Me-5), 24.2 (Me′-5), 15.4 (Me-3), 12.5 (Me-4); MS (70eV) m/z 69 (20), 79 (23), 93 (50), 107 (22), 121 (100), 136 (50), 150 (25), 167 (3), 236 (5), 303 (0.5, $M^{\cdot+}$); $[\alpha]_D$ -113.2 (c 0.68, $CHCl_3$); mp 63.1°C-64.5°C.

Obtention of pure ((1R,4R)-3,4,5,5-tetramethylcyclopent-2-en-1-yl)methyl acetate [(1*R,4R*)-**2**].

Potassium carbonate (165 mg, 1.2 mmol) was added to a solution of pure **5** (200 mg, 0.66 mmol) in MeOH (10 mL). The suspension was stirred for 4 h at room temperature. After this time, water (10 mL) was added, and the solution was extracted with EtOAc (2x10 mL). The combined organic layers were successively washed with saturated aqueous $NaHCO_3$ (5 mL), and brine (5 mL), and dried over anhydrous $MgSO_4$, to give 96 mg of pure (1*R*,2*R*)-**6** after evaporation of the solvent. The crude residue was dissolved in CH_2Cl_2 (15 mL) and triethylamine (0.23 mL, 1.60 mmol), a catalytic amount of 4-dimethylaminopyridine (DMAP), and anhydride acetic (0.12 mL, 1.20 mmol) were added at room temperature. The resulting solution was stirred for 4 h, then poured into water (10 mL) and extracted with CH_2Cl_2. The combined organic layers were successively washed with 1M aqueous HCl (5 mL), saturated aqueous $NaHCO_3$ (5 mL), and brine (5 mL), and dried over anhydrous $MgSO_4$. The solvent was removed under reduced pressure, and the resulting crude residue was purified by flash column chromatography on silica gel, using a 98:2 mixture of hexane and Et_2O as eluent, to give 105 mg (80% yield) of acetate (1*R,*4*R*)-**2** as a yellowish oil, which spectroscopical data were fully coincident with those described in the literature.[11] $[\alpha]_D$ -118.0 (c 0.1, $CHCl_3$)

^{1}H NMR spectrum of **7**.

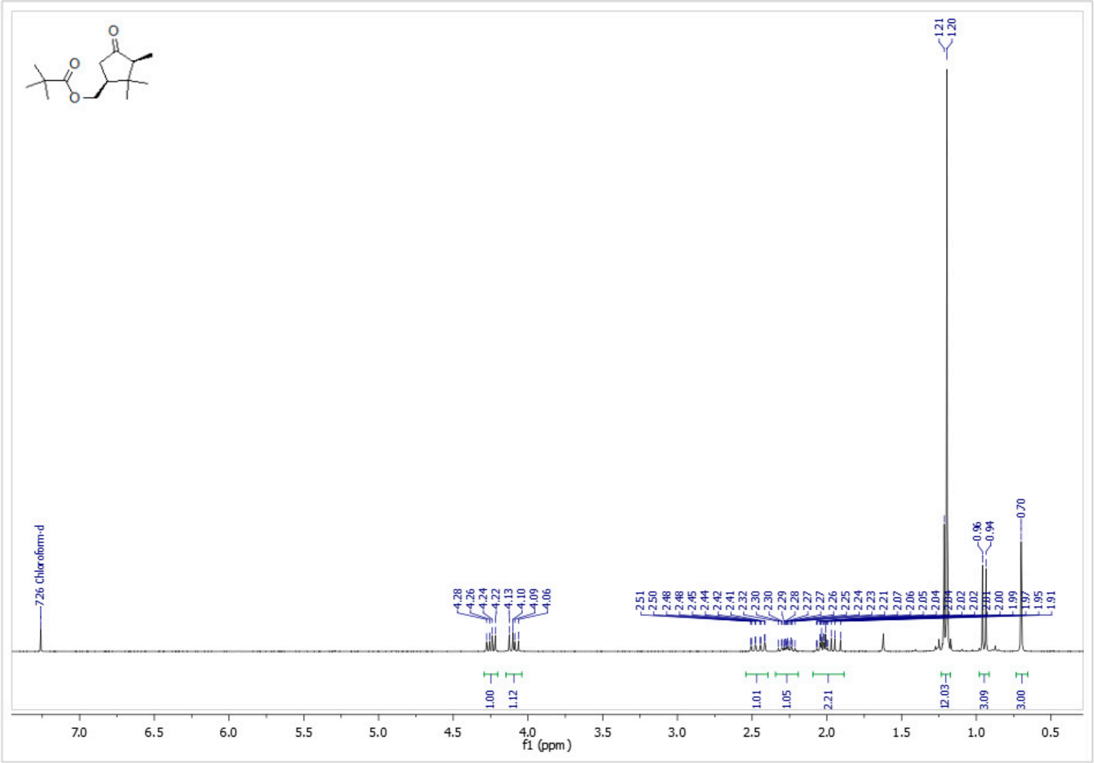

^{13}C NMR spectrum of **7**.

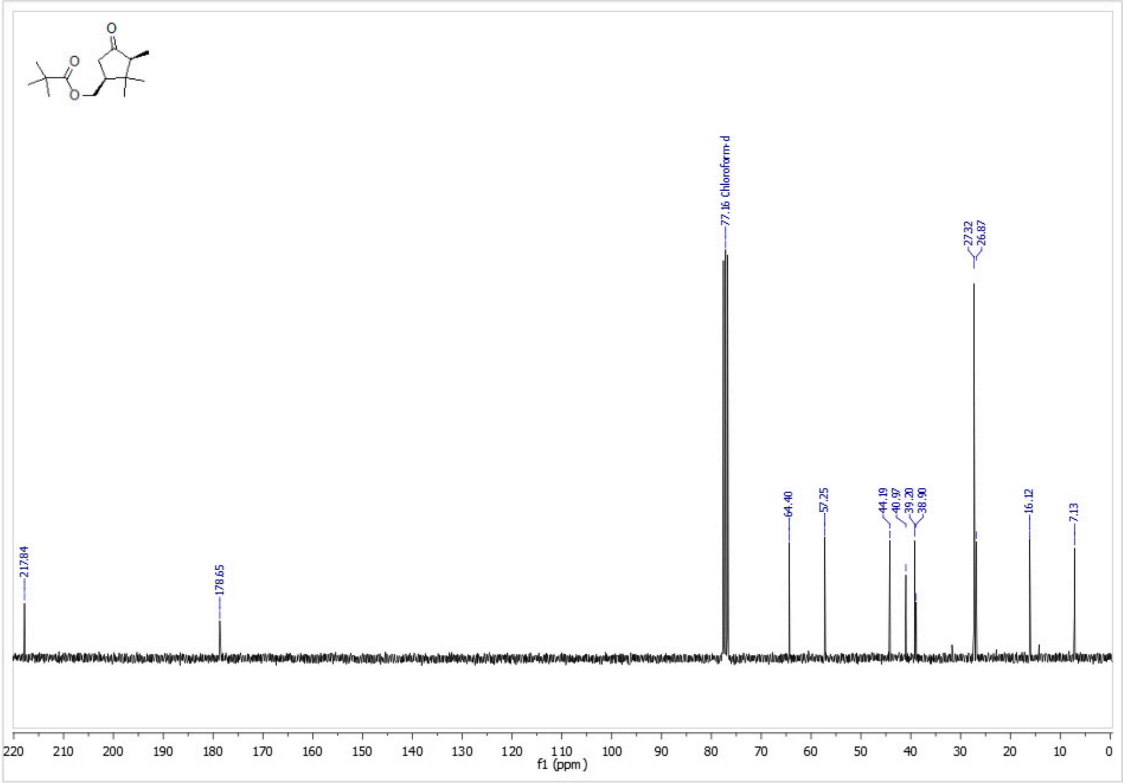

^{1}H NMR spectrum of **8**.

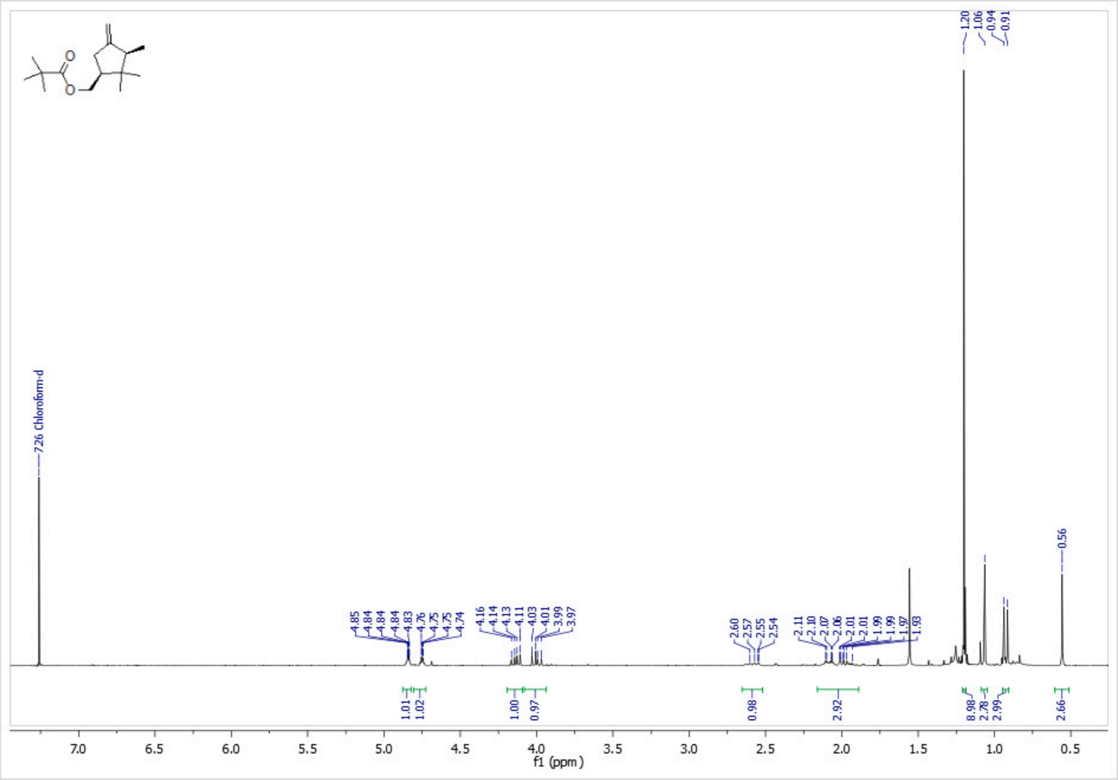

^{13}C NMR spectrum of **8**.

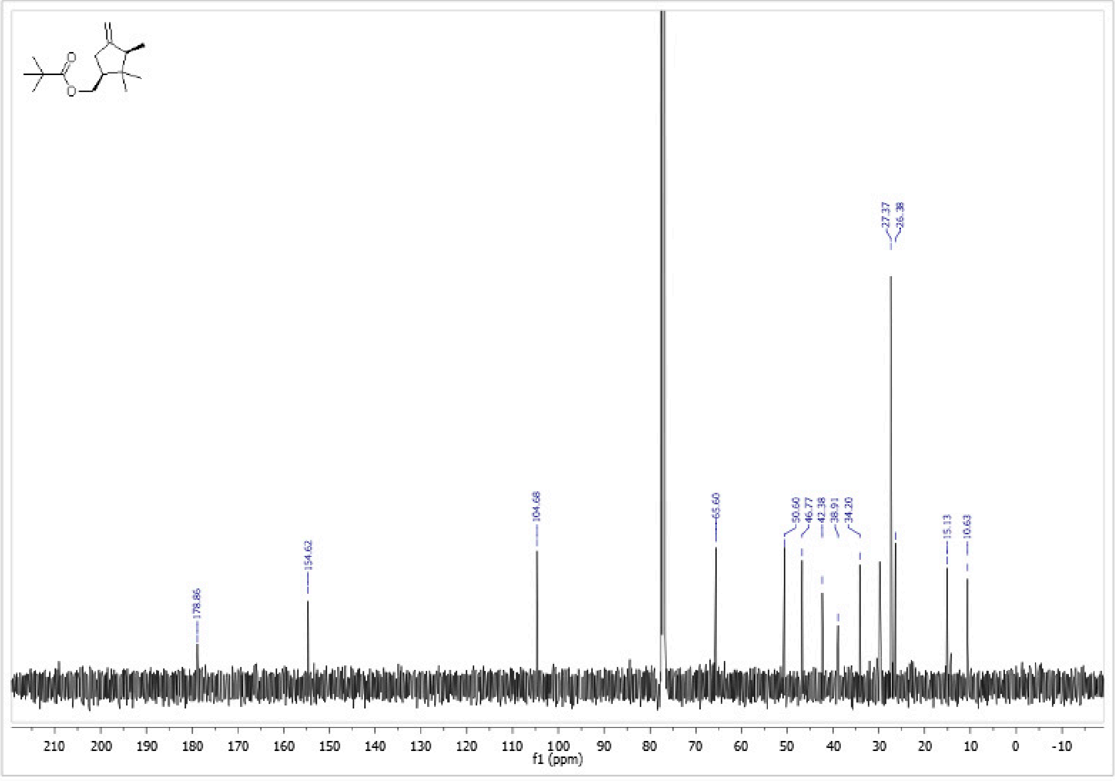

^{1}H NMR spectrum of (±)-(*cis*)-**3**.

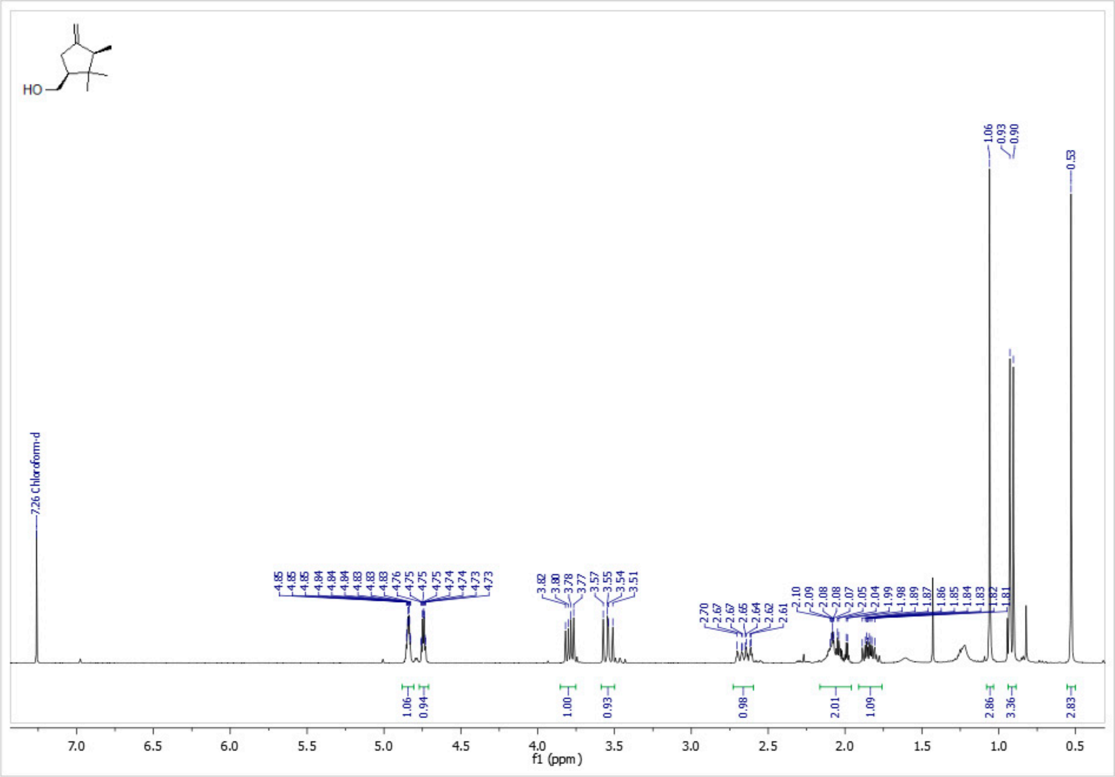

^{13}C NMR spectrum of (±)-(*cis*)-**3.**

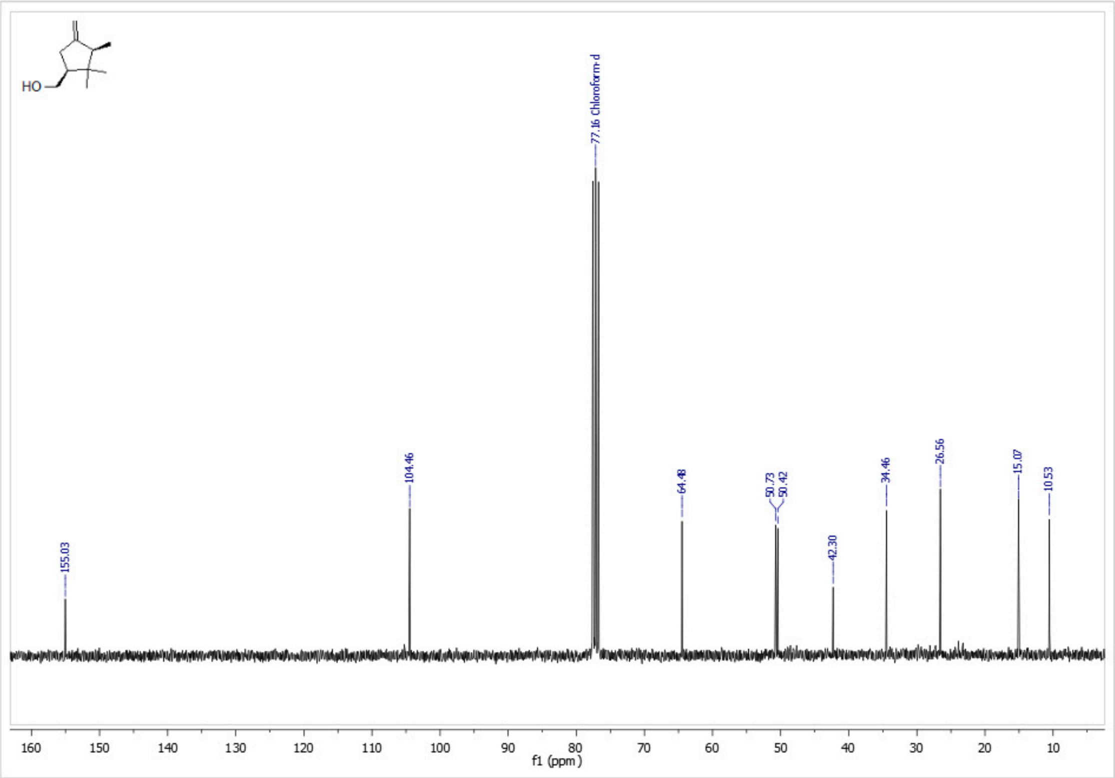

^{1}H NMR spectrum of (±)-(*cis*)-**6**.

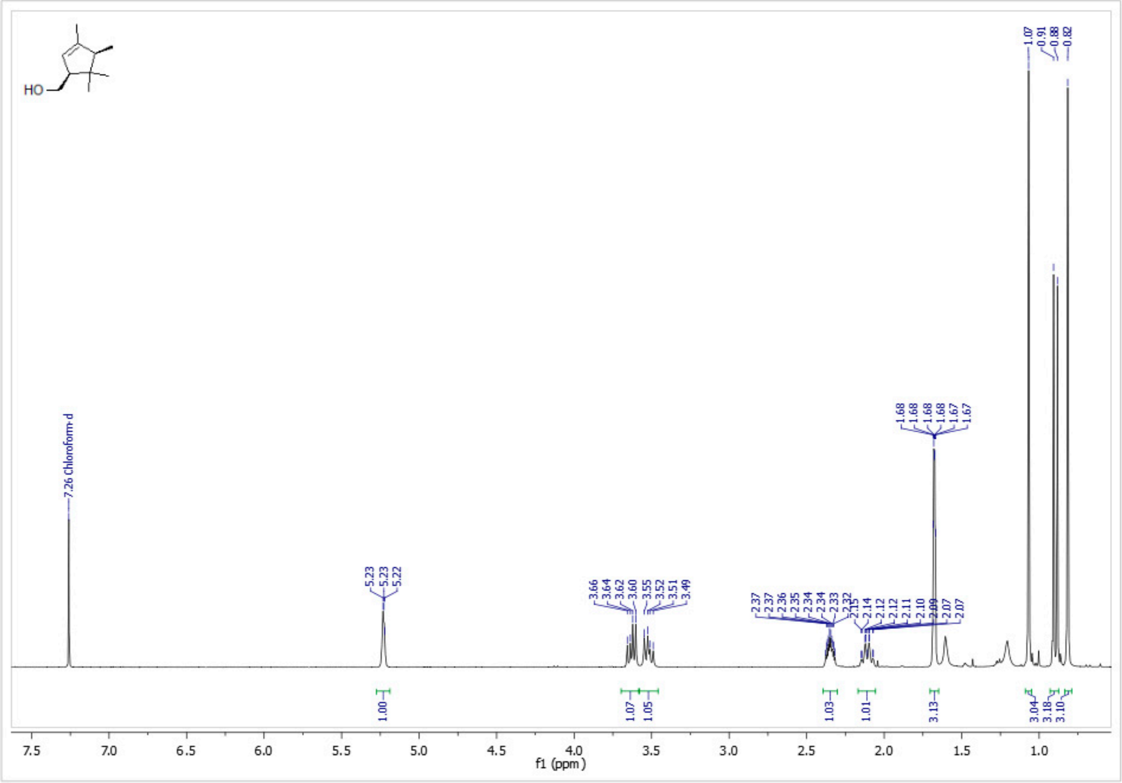

^{13}C NMR spectrum of (±)-(*cis*)-**6**.

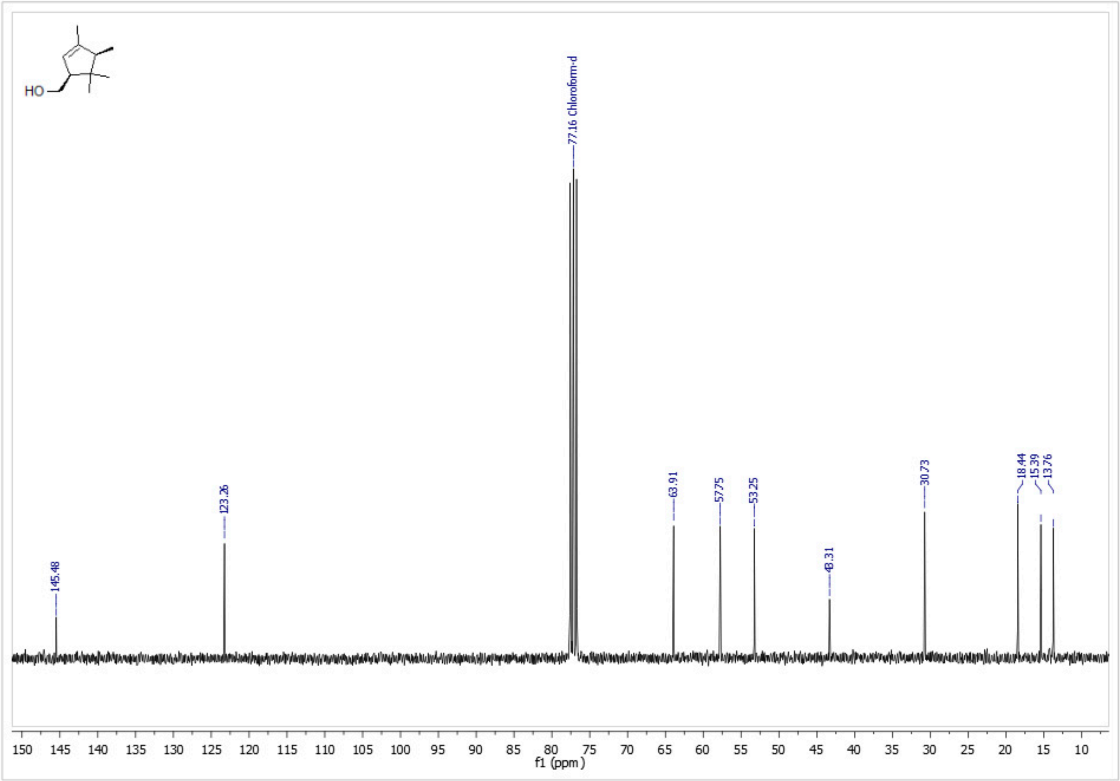

^{1}H NMR spectrum of (±)**-(cis)-2**.

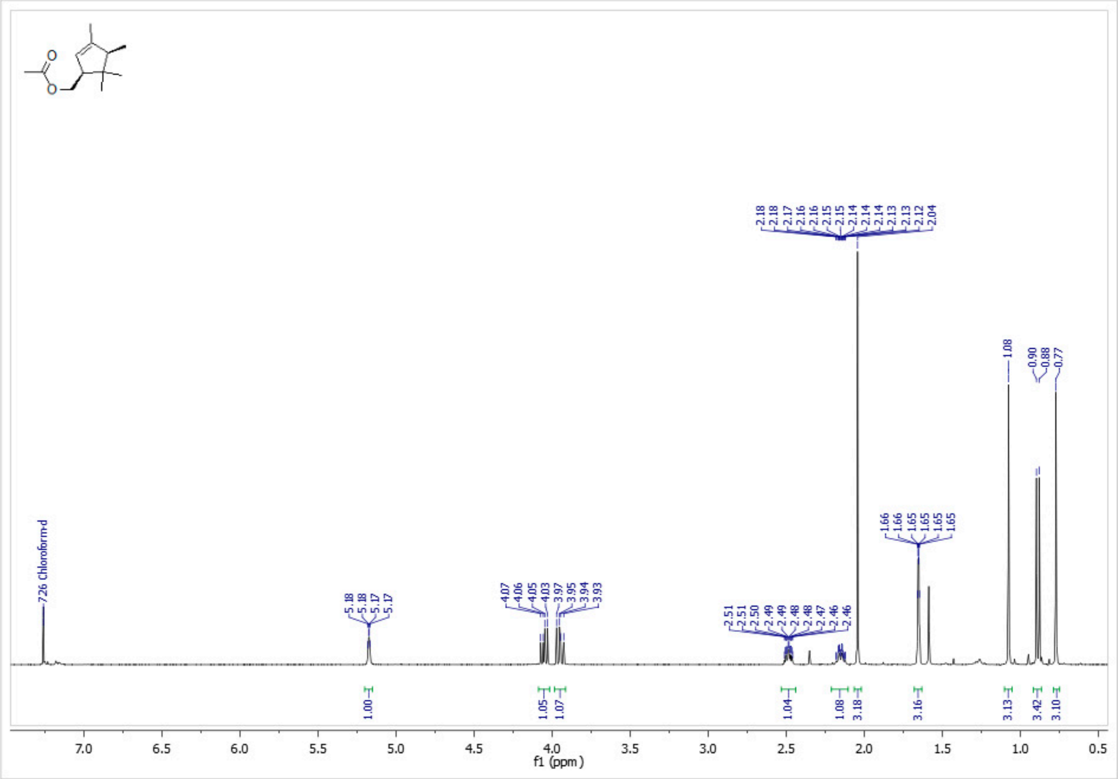

^{13}C NMR spectrum of (±)**-(cis)-2**.

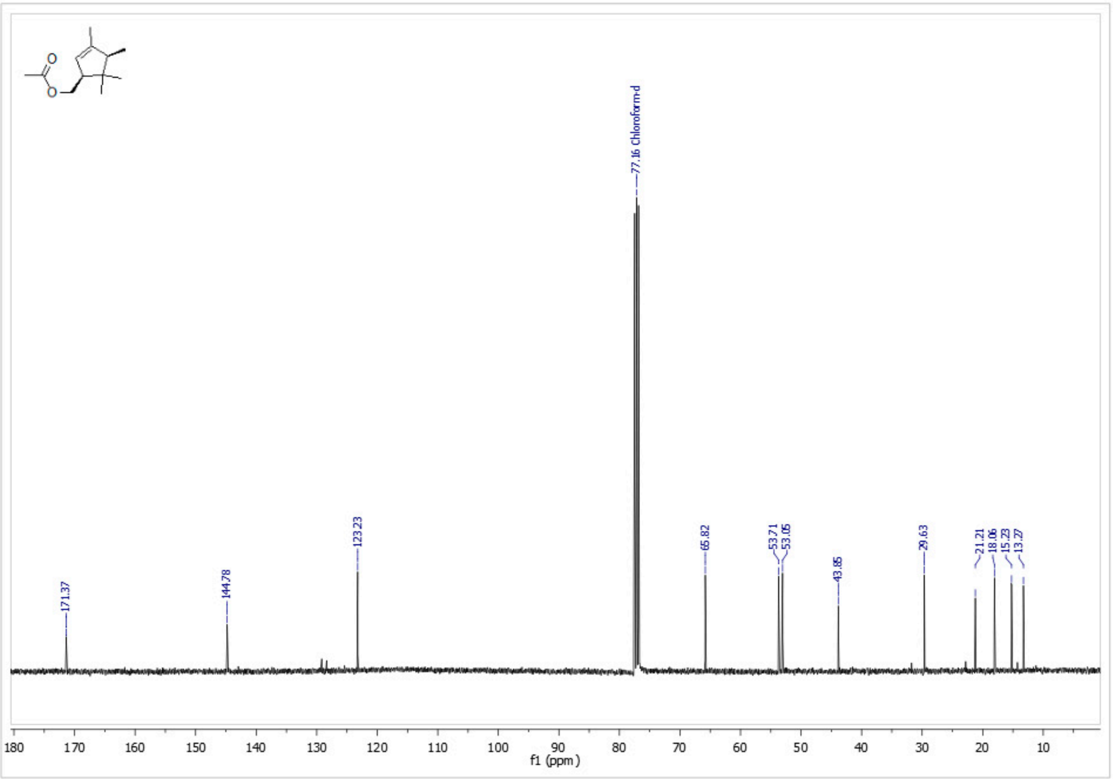

^{1}H NMR spectrum of **9**.

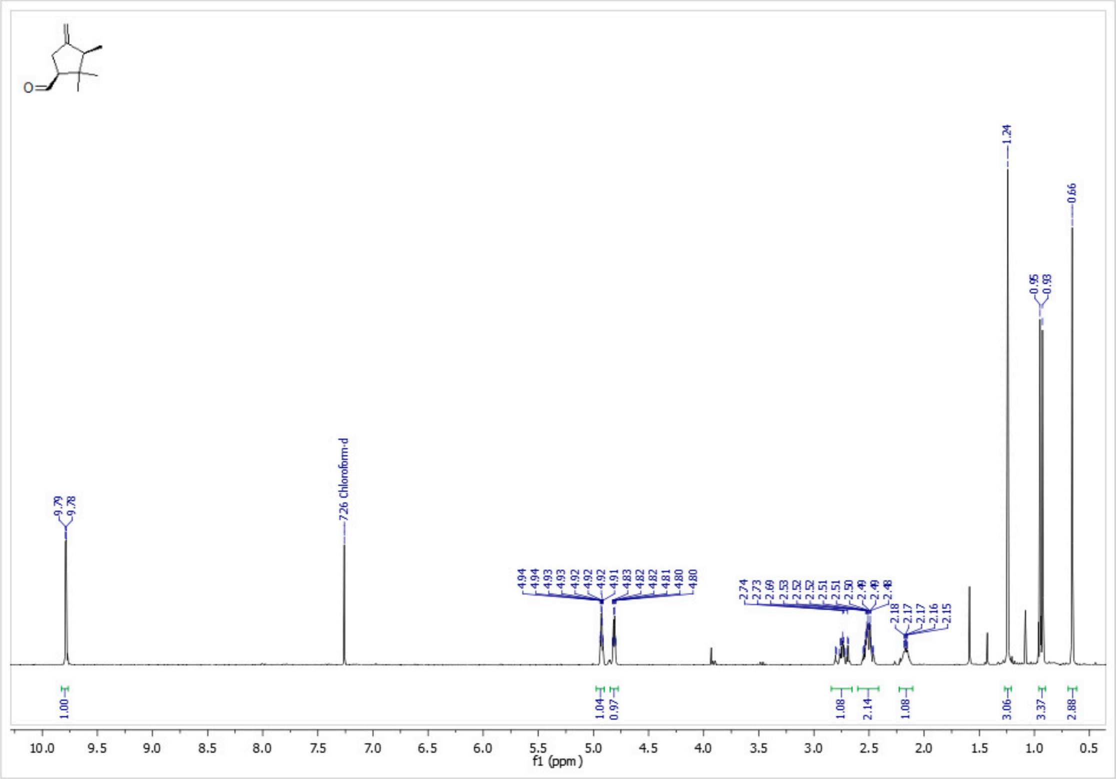

^{13}C NMR spectrum of **9**.

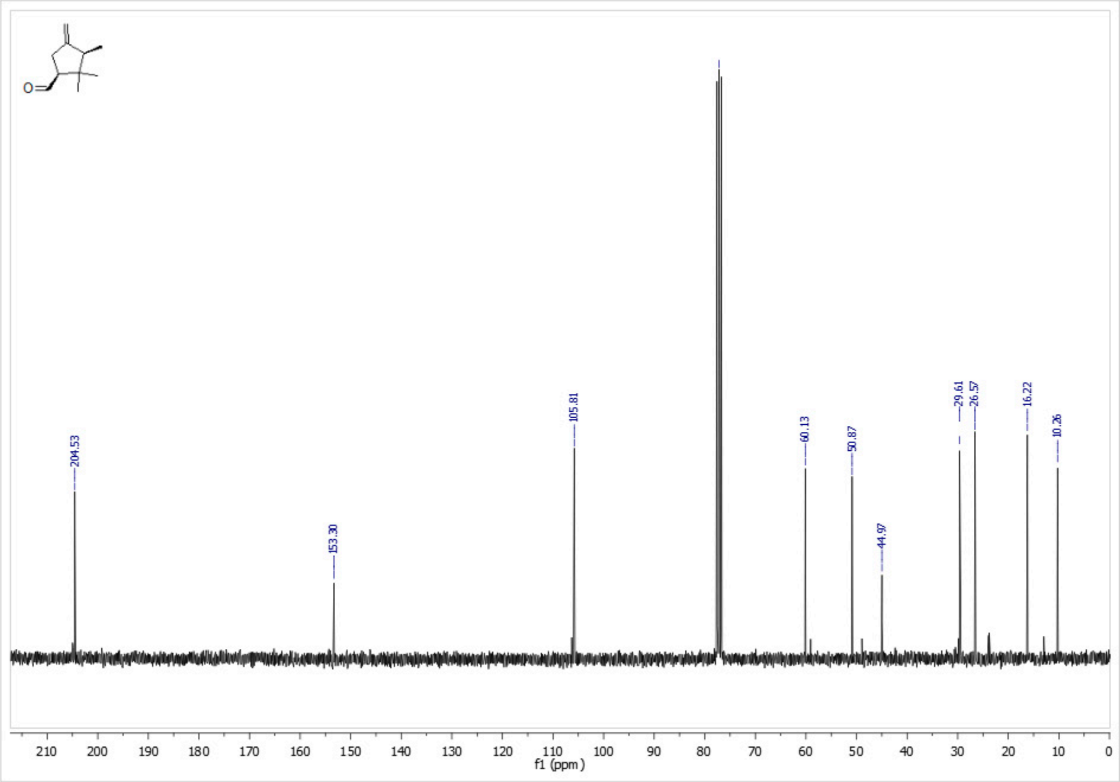

^{1}H NMR spectrum of **10**.

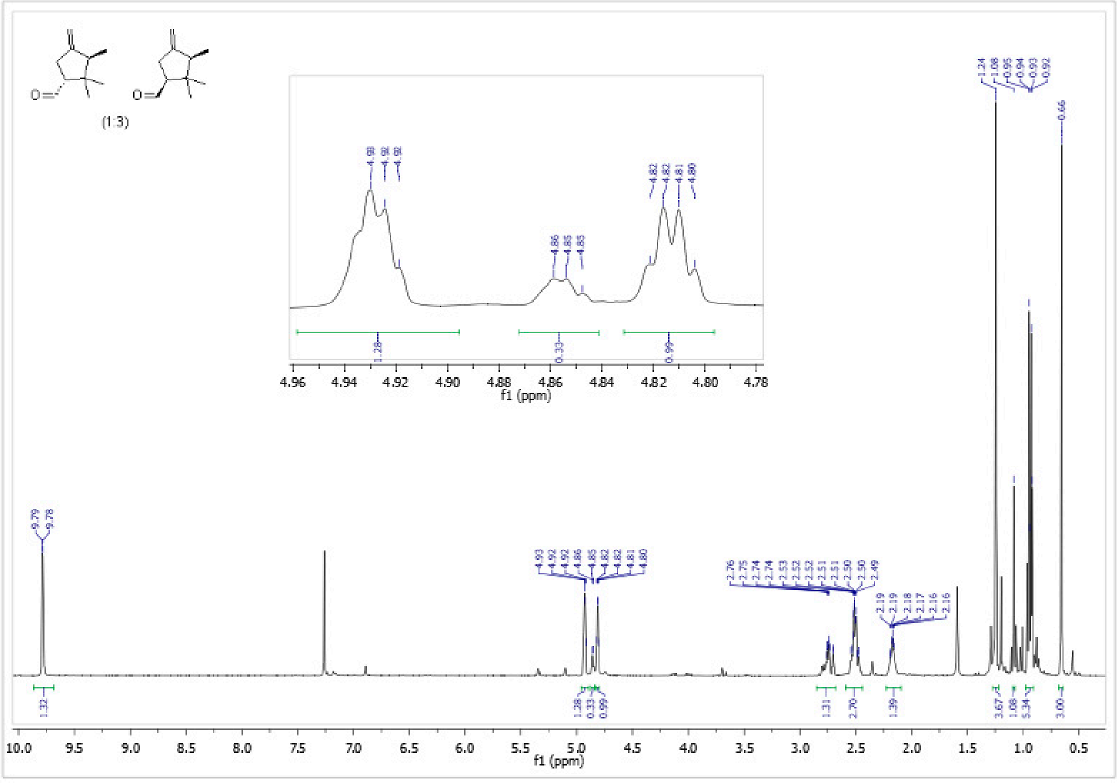

^{1}H NMR spectrum of (±)-(*trans*)-**6**.

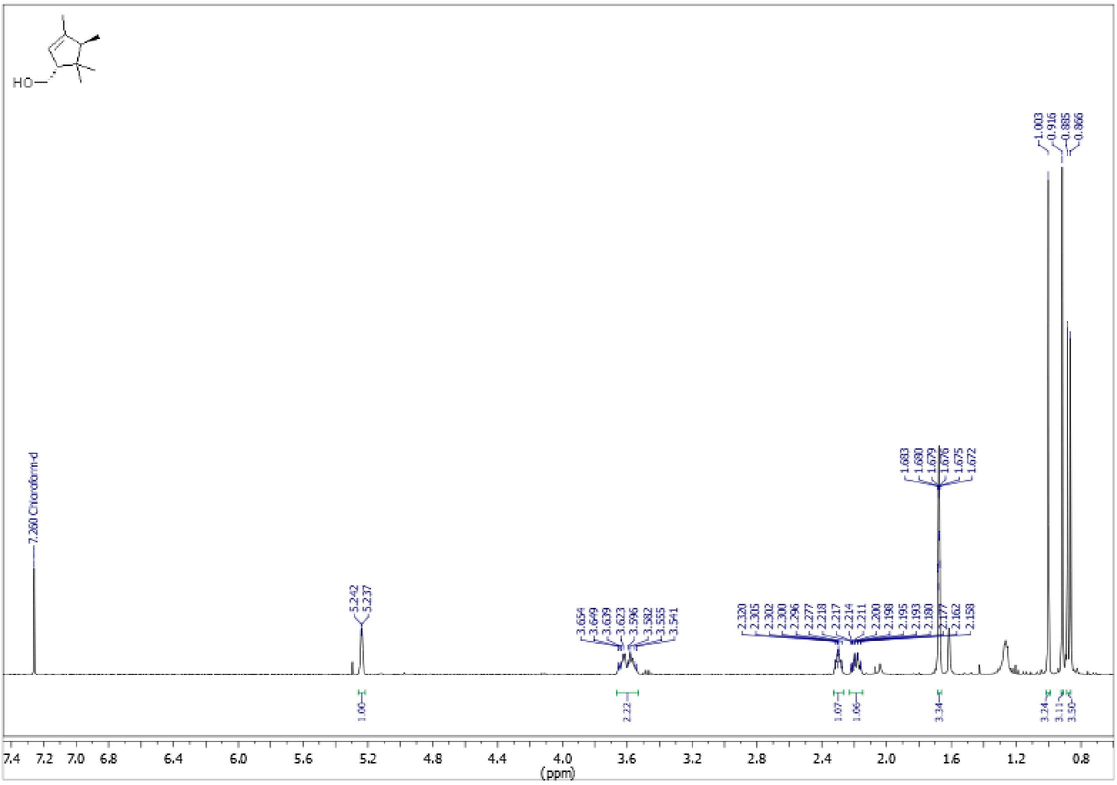

^{13}C NMR spectrum of (±)-(*trans*)-**6**.

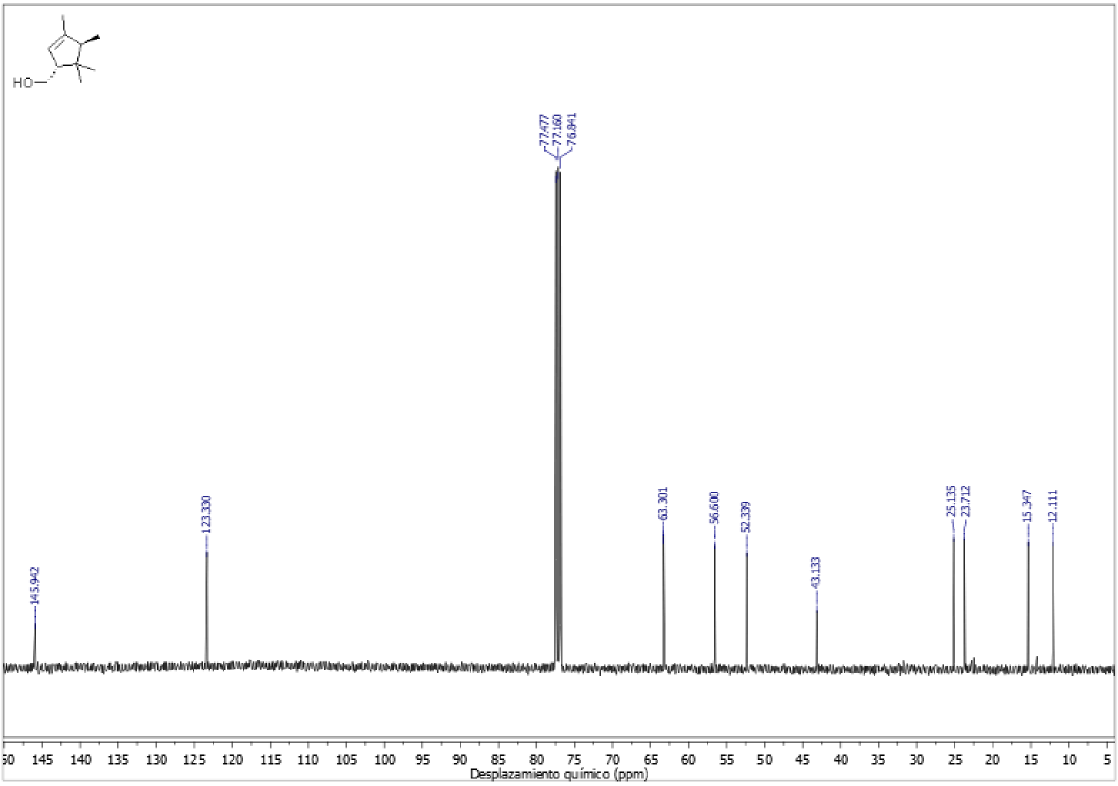

^{1}H NMR spectrum of (±)**-(trans)-2**.

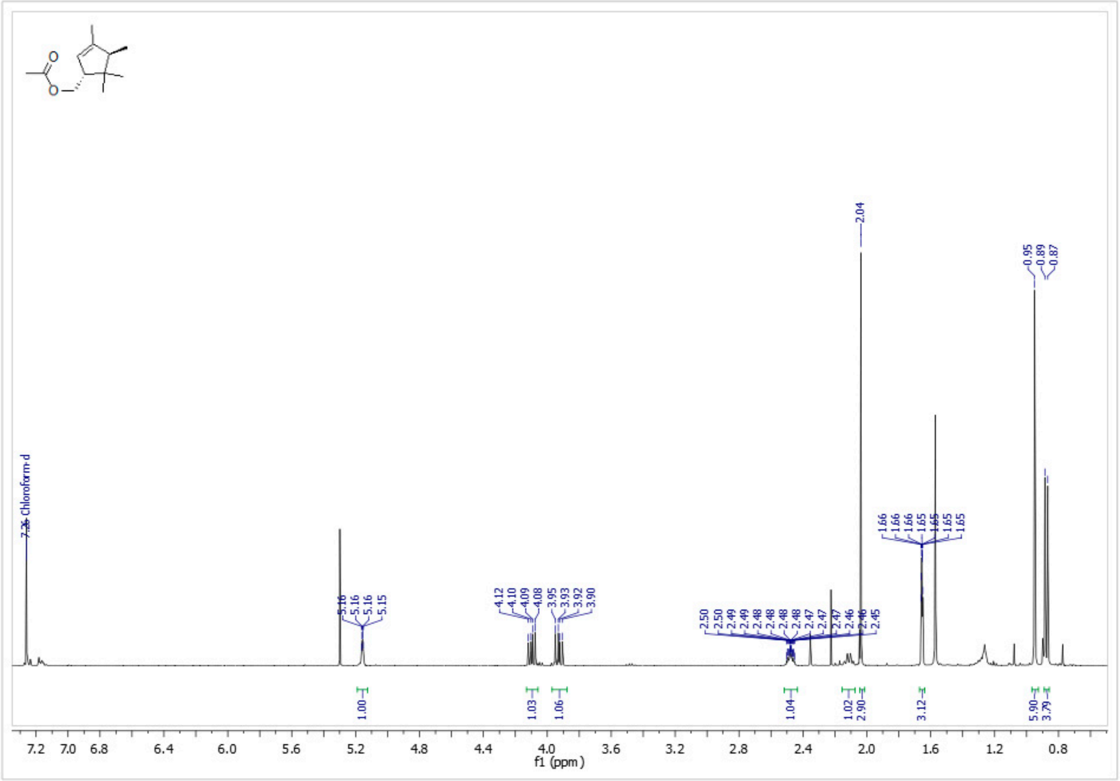

^{13}C NMR spectrum of (±)-**(trans)-2**.

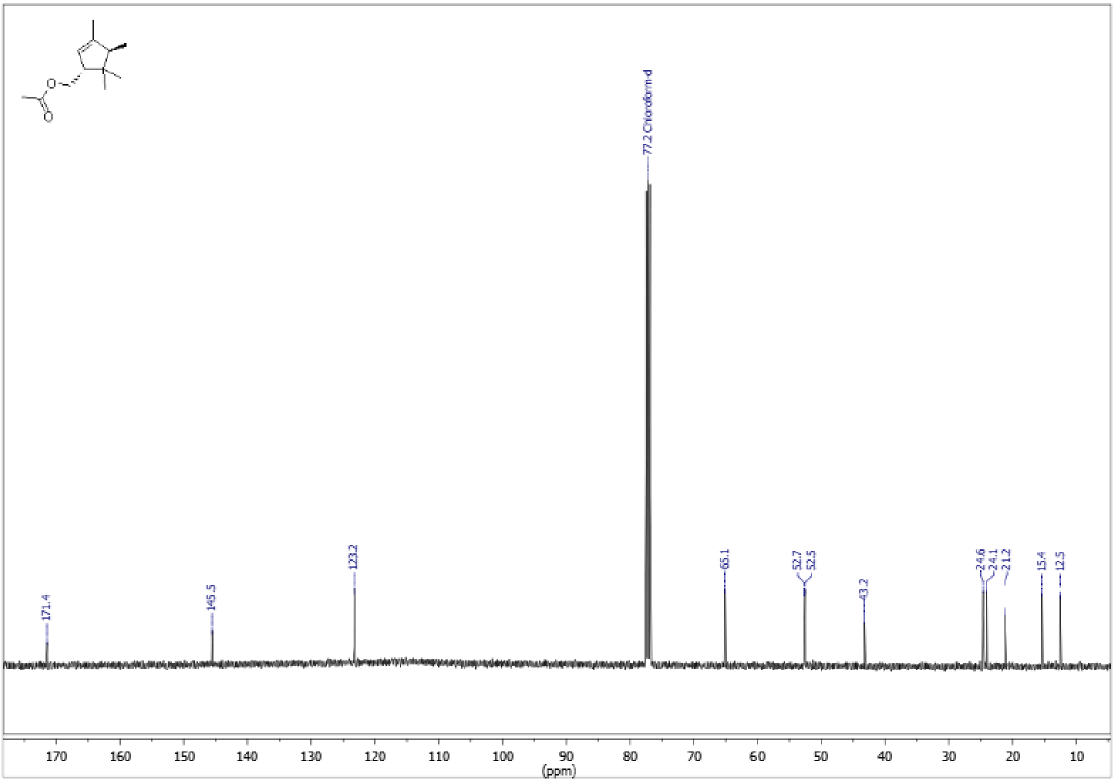

^{1}H NMR spectrum of **5**.

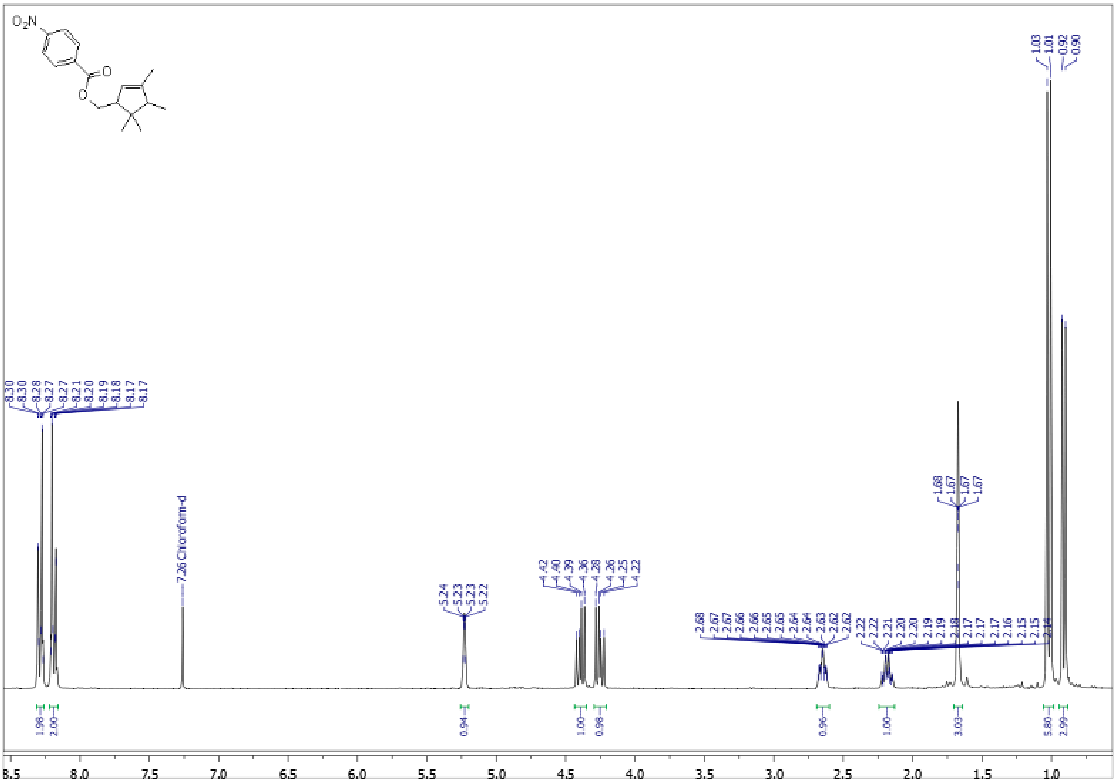

^{13}C NMR spectrum of **5**.

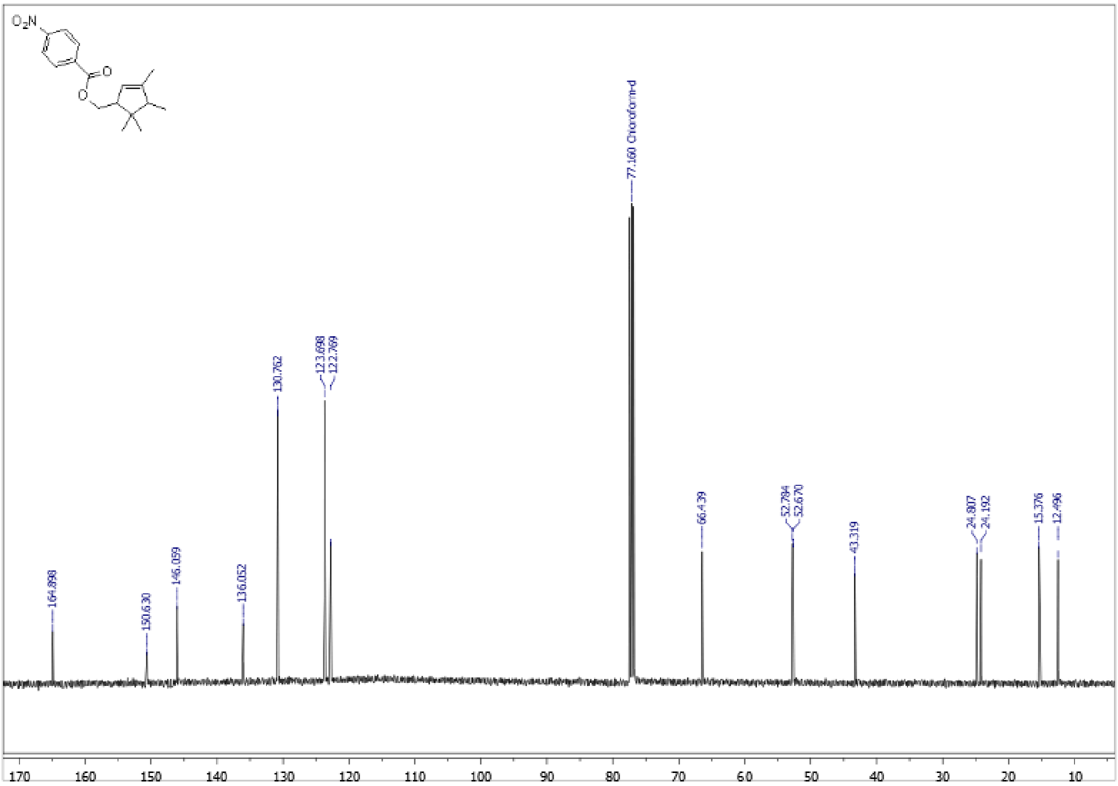

^{1}H NMR spectrum of (1*R*,4*R*)-**2**

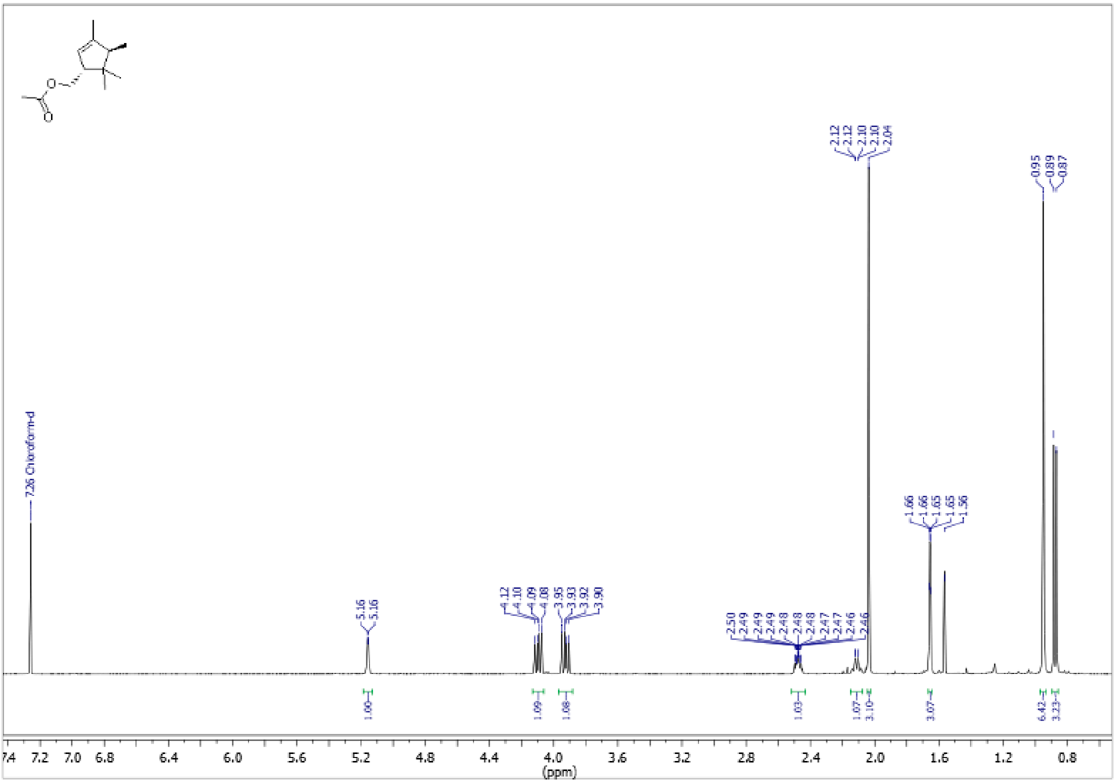

^{13}C NMR spectrum of (1*R*,4*R*)-**2.**

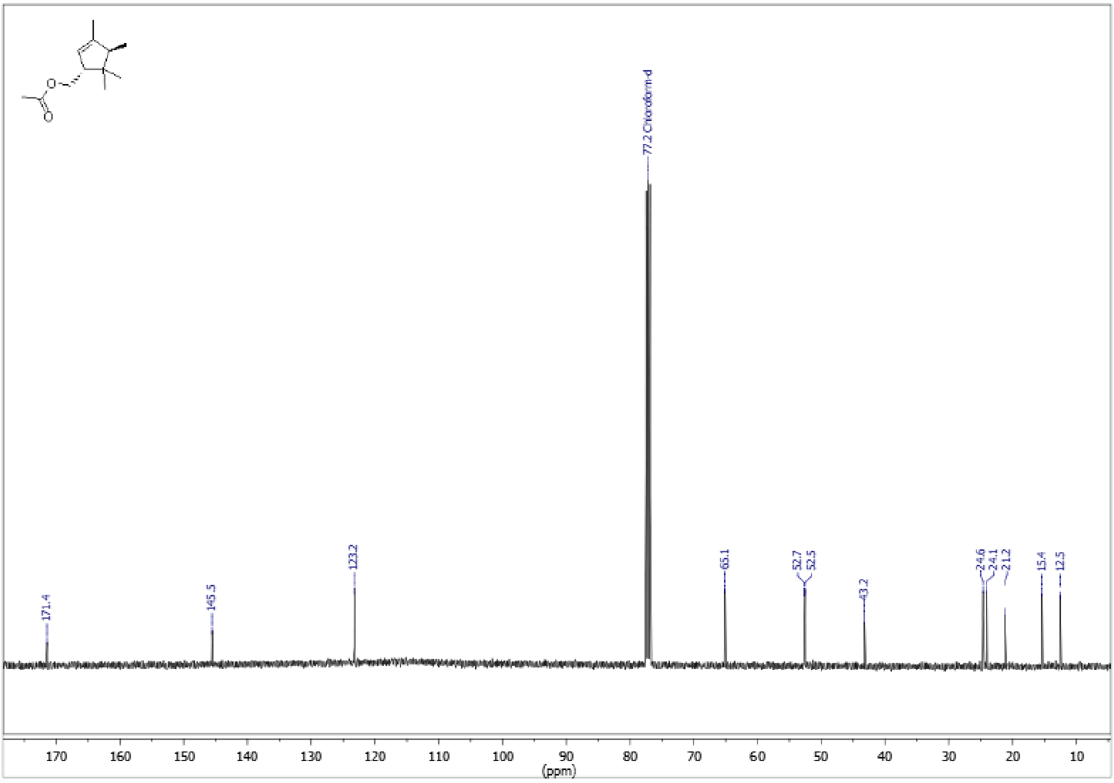

Figure S3. Mass spectra fragmentation observed for samples of (*trans*)-α-necrodol obtained by hydrolysis of the minor sex pheromone component of D. aberiae virgin female volatile collections (A), synthetic *β*-necrodols (±)-(*cis*)-3 or (±)-(*trans*)-3 (B) and γ-necrodol (C).

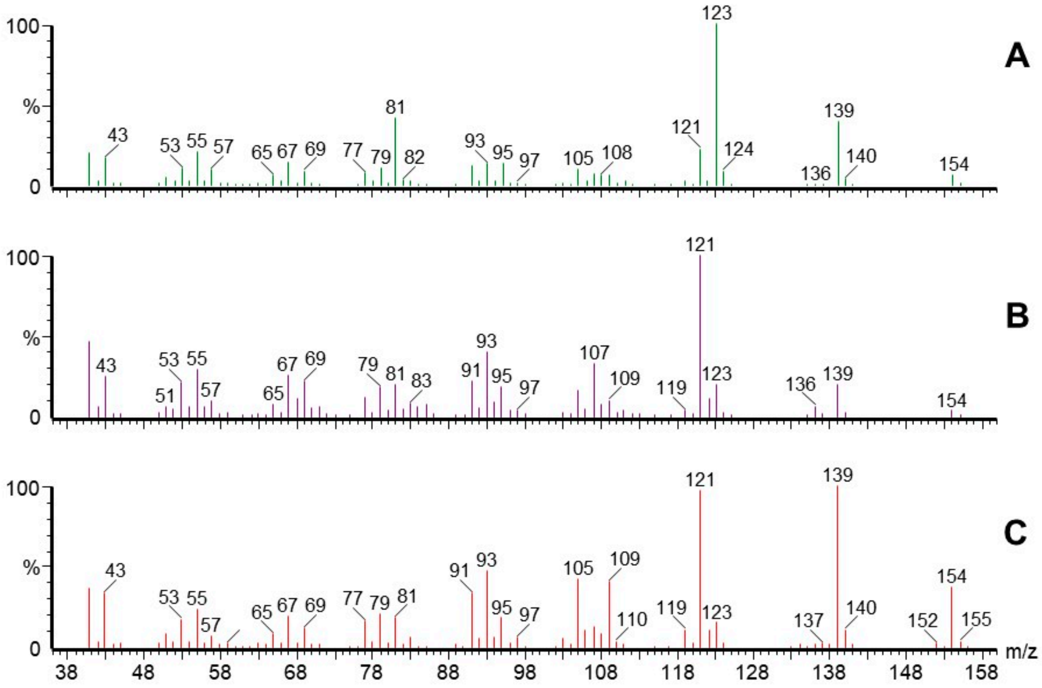

Figure S4. Gas chromatography (GC) with chiral stationary phase column of purified (1*R*, 4*R*)**-6,** (±)-(*cis*)**-6 and** (±)-(*trans*)**-6.** Cromatography conditions: BETA DEX 120 chiral capillary column (30 m × 0.25 mm i.d. × 0.25 µm; SUPELCO) installed in a GC Focus series instrument (Thermo Scientific., Milan, Italy) equipped with a flame ionization detector (FID) set at 250 °C. The GC oven temperature was raised at 0.6 °C/min from 50 to 115 °C and then at 25 °C/min to 150 °C, which was finally held for 10 min. Carrier gas was nitrogen at 1 mL/min flow rate.

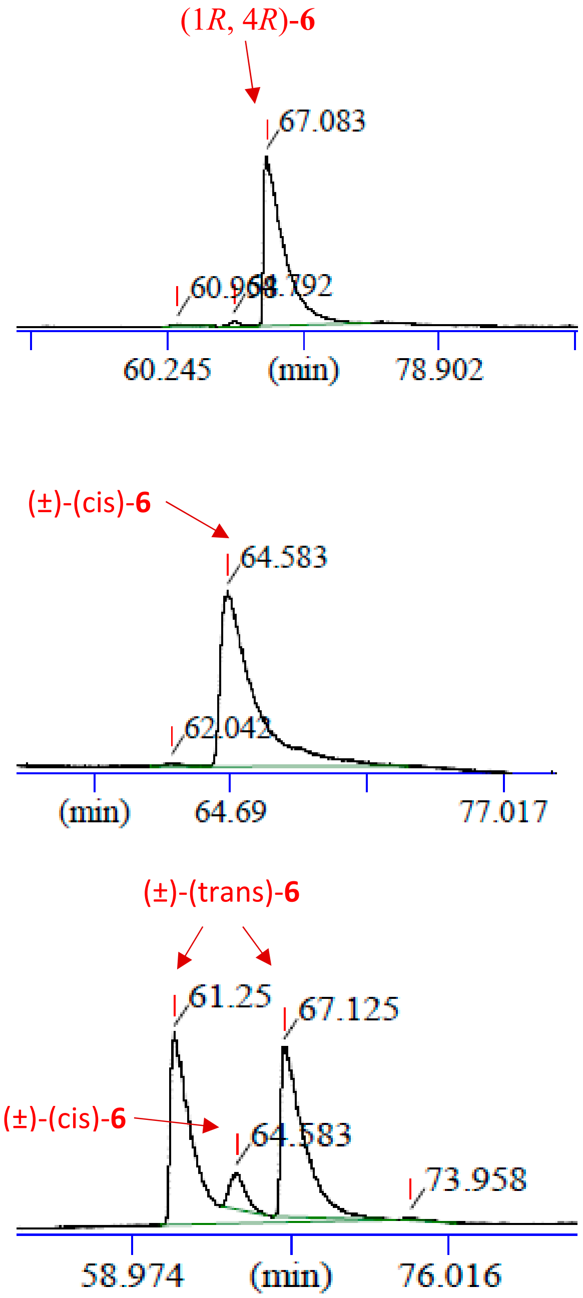

Supplement: Supplementary file 1 [file insects-16-00318-s001.zip › insects-3496941-supplementary.pdf]
